# Morphology characters of resected femoral and tibial surface in chinese population: intraoperative anthropometric study in patients at a tertiary hospital

**DOI:** 10.1186/s12893-022-01584-1

**Published:** 2022-04-19

**Authors:** Yiming Xu, Bin Feng, Yulei Dong, Zhibo Zheng, Yanyan Bian, Xisheng Weng

**Affiliations:** 1 Department of Orthopedics, Peking Union Medical College Hospital, Chinese Academy of Medical Sciences & Peking Union Medical College, No.1 Wangfujing Shuaifuyuan, Dongcheng District, Beijing, 100730 China; 2 Department of International Medical Services, Peking Union Medical College Hospital, Chinese Academy of Medical Sciences & Peking Union Medical College, Beijing, 100730 China; 3 State Key Laboratory of Complex Severe and Rare Diseases, Peking Union Medical College Hospital, Beijing, 100730 China

**Keywords:** Total knee arthroplasty, Anthropometry, Tibia, Femur, Prothesis

## Abstract

**Backgrounds:**

Mismatch between knee surface and prosthesis components is related to postoperative complications. Morphological differences between ethnicity and gender may affect prosthesis coverage. The purpose of this study is to describe morphological characters of resected knee surface (distal femur, proximal tibia) in the Chinese population, analyze the influence of gender and other demographical factors, and validate the effect of ethnic difference by calculating the coverage of Western-designed knee prostheses on Chinese knee surface.

**Methods:**

Intraoperative anthropometries were performed during total knee arthroplasty performed by one single team. After screening out severe deformities and bone defects, data were separated via prosthesis system. Multiple linear regression and partial correlation analysis of morphological parameters on age, gender, height, weight were used to find out independent factors influencing morphology. Based on the 5 mm-tolerance in the prosthesis, simulation on scatter plots was brought out to calculate the prosthesis coverage to the resected bone surface.

**Results:**

A total of 865 cases of total knee arthroplasty were involved in this study. Though gender differences were found in all knee morphological parameters regardless of the type of prosthesis, significant association was only found between gender and mediolateral width of femoral surface after adjusting demographical factors (p < 0.001). The two included prosthesis systems, Genesis-II and Scorpio NRG covered most cases in at least one dimension. Males had lower complete coverage and higher no coverage rate on femurs. Asymmetry prostheses had higher lateral coverage on tibiae.

**Conclusions:**

Based on our analysis, the only confirmed demographical factor in knee morphology is gender on femoral mediolateral length. Wider femoral prostheses for males may improve results of gender-specific prostheses. The overall fitness between Western-designed prostheses and Chinese knee surface is appliable, but the ratio of complete coverage is low. Further modification of prostheses systems can aim at the number of sizes and geometrical shapes.

## Introduction

Suitable coverage of prosthesis is essential for achieving a successful outcome in total knee arthroplasty (TKA) [1]. Modern TKA systems have a series of prosthesis sizes to improve coverage, but there are still some unfit cases. Previous studies have demonstrated that overhang or under-coverage of the prosthesis, especially in tibial plateau, could result in severe complications like pain, dyskinesia, bleeding, and loosening [2–4]. Since the appearance of knee arthroplasty, researchers started measurement of knee joint morphological characters to get accurate data for prosthesis design [5–7]. Based on their achievements, modern anatomic condylar TKA systems contained a precisely designed series of sizes of prostheses for different sizes of knee surfaces. However, these studies were mainly conducted in Caucasians, lacked participants from Asian populations and other ethnicities [8]. Systematic reviews revealed the ethnic difference of knee morphology [9]. Measurements also found mismatch between knee prosthesis and Asian knees [10, 11]. Gender has been recognized as another factor influencing knee morphology. In any given anteroposterior length, both Asian and Caucasian women have smaller femoral mediolateral width than males, indicating that females might suffer from mediolateral overhang with a conventional prosthesis [12–15]. So narrower femoral prostheses were designed for females to improve it [16]. According to follow-up results, no gender difference was identified in conventional prosthesis and gender-specific systems presented no benefit in function [17, 18]. Someone believed that the morphological gender difference was influenced by other factors like height and weight [16].

Anthropometry measured human anatomic structures with given standards to describe their morphological characters. In the early time, forerunners performed anthropometric measurements mainly with X-ray photography or cadaver [5–7]. The accuracy was influenced by photo distortions or formalin procession. Some recent researches used tomography for exact measurement, but these virtual assessments could not reveal the actual situation after osteotomy [8, 13, 19, 20]. Some surgeons conducted intraoperative anthropometry [11, 21–23]. But the sample size was small and the evaluation methods of prosthesis coverage were variable and could not fit practical truth. In this study, we carried out an intraoperative anthropometric study based on patients receiving TKA in our surgical team. With statistical analysis and measurements of scatter plots developed from previous studies, we aimed to evaluate the influence of gender and other factors in morphology of resected knee surfaces, and compare the coverage of modern Western-designed knee prostheses in Chinese population.

## Materials and methods

### Intraoperative anthropometry

**Fig. 1:**
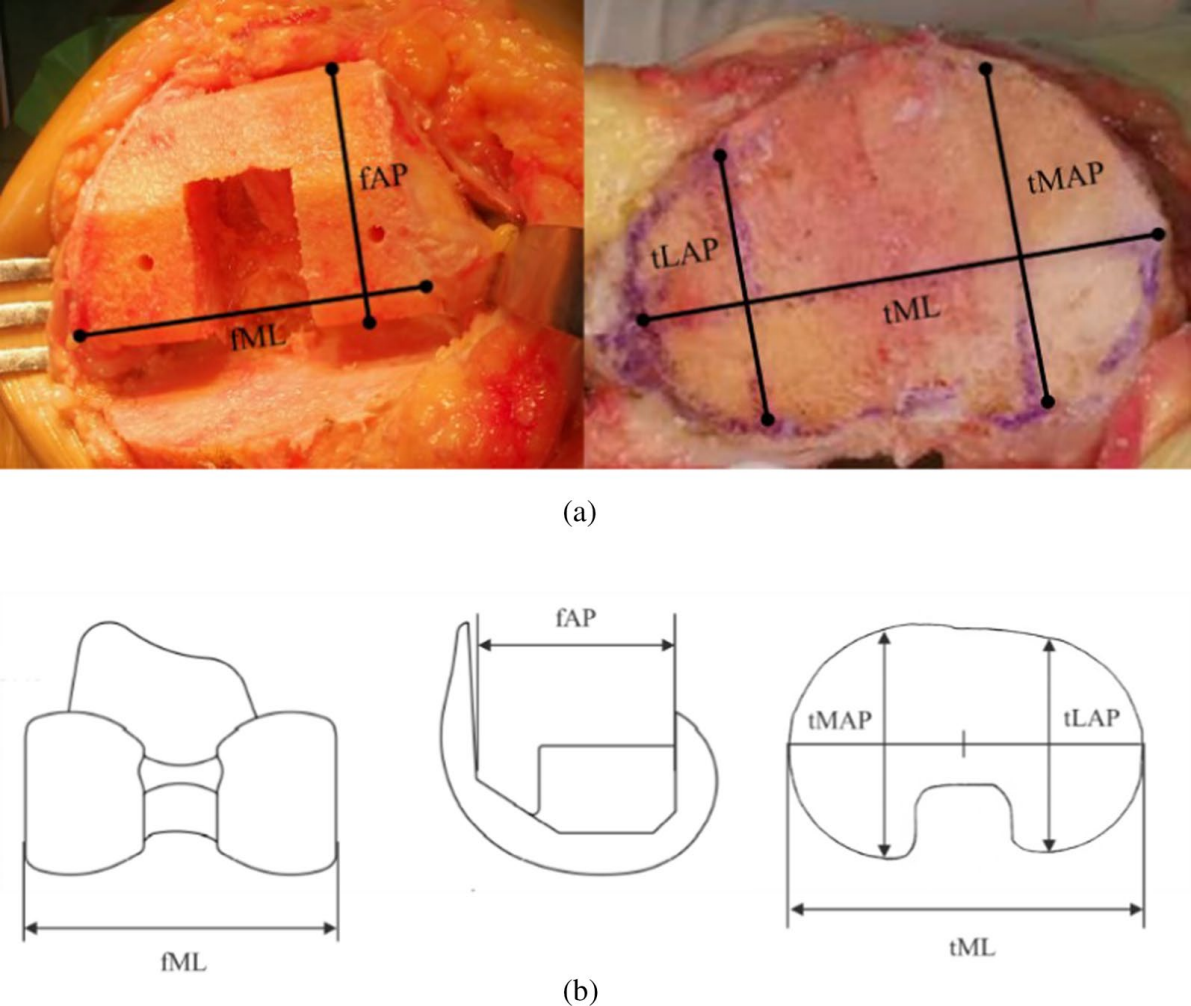
Diagram of the measurement of anthropometric parameters on (**a**) resected surface of femur and tibia (**b**) femoral and tibial test model

This study was approved by the Institutional Review Board at Peking Union Medical College Hospital. All included patients signed informed consent statements before operation. Chinese patients who underwent primary TKA by our surgical team were included. Cases were excluded if there was serious valgus or varus requiring augmentation, or bone defects disrupting bony sign that the measurement could not be conducted. TKA operations were conducted by the same team under general anesthesia. The knee joint was exposed through a parapatellar approach. The femoral and tibial osteotomies were performed with an oscillating saw according to the TKA system surgical technique handbook. Then anthropometric parameters were measured with a sterile slide caliper in millimeters three times. The average result was recorded, retaining two decimal places. Femoral parameters contained the anteroposterior length (fAP) and mediolateral width (fML) of resected surface (Fig. 1a). The mediolateral width (tML) was also measured in the tibial resected surface. For tibial anteroposterior length, two parameters were applied: medical plateau length (tMAP) was the anteroposterior length of the vertical line passing medial quadrant point of mediolateral width, while lateral plateau length (tLAP) was the length of the similar vertical line passing lateral quadrant point of width (Fig. 1a). The corresponding parameters of prostheses were measured from different sizes of test models in the same way (Fig. 1b). Patient demographic data such as gender, age, body mass index (BMI), and the type of TKA system were assessed from the medical record.

### Prosthesis coverage simulation

**Fig. 2:**
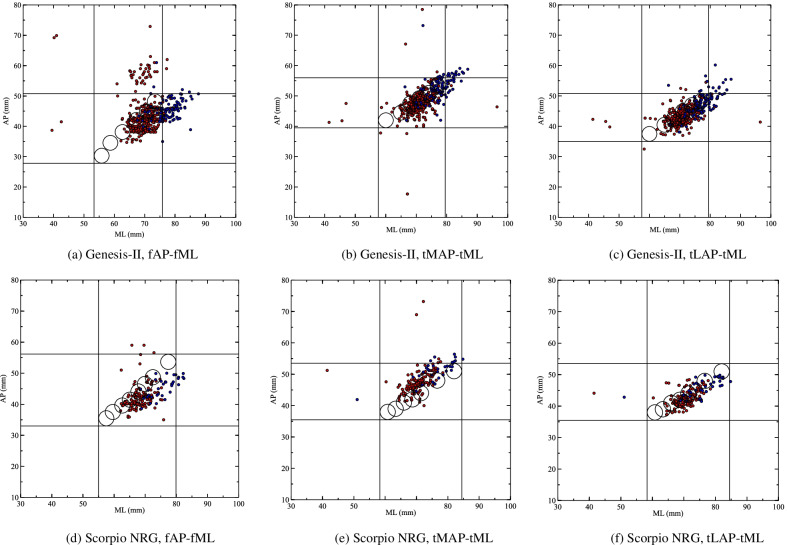
For Genesis-II and Scorpio NRG cases, (**a**, **d**) are femoral anteroposterior length (fAP) versus femoral mediolateral width (fML), (**b**, **c**, **e**, **f**) are tibial medial or lateral anteroposterior length (tMAP or tLAP) versus tibial mediolateral width (tML). Male cases are in blue and female cases are in red. Circles and tangents are drawn for evaluation of prosthesis coverage

In the early development of condylar knee prothesis, Erkman et al. illustrated that two prostheses with a size difference of 5 mm covered most patients, so ± 2.5 mm has been regarded as the maximum tolerance of prosthesis [6]. This threshold was inherited by Cheng et al. and performed well in our surgical practice. In this study, we conducted measurements on scatter plots to evaluate prosthesis coverage. These measurements were developed from Cheng’s method [24]. The anthropometric data of TKA cases were plotted in the coordinate system, with mediolateral (ML) width as the x-axis and anteroposterior (AP) length as the y-axis. Then circles with 2.5 mm radius were plotted in the same system and the centers were determined by corresponding parameters of different sizes of prostheses. The vertical and horizontal tangents of each circle were also plotted. The degree of coverage was defined into three levels. “Complete coverage” referred to case points that fell into any circle, indicating that both mediolateral and anteroposterior parameters were covered. “Relative coverage” referred to case points not included by any circle but lied in the cross formed by tangents, indicating that one dimension was covered. “No coverage” referred to case points outside the cross described above, indicating that no dimension was covered. Because the cross made by tangents of each circle had overlapping areas, only the tangents forming the border of the cross-region were shown in plots (Fig. 2).

### Statistical analysis

Comparisons between different gender were conducted via an independent t-test. Stepwise linear regression and partial correlation analysis were performed to determine the influence of gender, height, and weight on the prediction of morphological parameters. As the only categorical variable, gender was transformed into 1 (for male) or 0 (for female) for analysis. The prosthesis coverage was evaluated with the percentage of the covered point at different levels. All statistical analysis was performed via SPSS Statistics 19.0 (SPSS Inc, Chicago, IL, USA). The numerical data obtained were summarized in tables as the arithmetic mean ± standard deviation. The nominal and ordinal data were evaluated as frequency and percentages. The level of significance was defined at p = 0.001 and all numerical data retained two decimal places.

## Results

### Demographic data

This study identified 871 TKA cases between October 2011 and December 2019. Three cases were excluded because of bone defects and another three were screened out due to severe knee deformity. The average age of the remaining 865 cases was 66.93 ± 7.99 years old, and the average body mass index (BMI) was 26.90 ± 3.64. Female patients (690 cases, 79.77%) were much more than males (175 cases, 20.23%). Osteoarthritis (804 cases) took the majority of diagnoses (92.95%). There were also 59 cases (6.82%) of rheumatoid arthritis and 2 cases (0.23%) of Sjogren’s syndrome. The most popular TKA systems used in this study were Genesis-II (Smith & Nephew, 505 cases), Scorpio NRG (Stryker, 171 cases), and NexGen LPS (Zimmer, 111 cases).

### Characters influencing knee morphology

Because the osteotomy was performed by specifically-designed templates provided by prosthesis systems, the knee anthropometric parameters were grouped by TKA systems for analysis (Table 1). The thick of resected bone no statistically gender difference, convincing the consistency of surgical procedures. In the primary analysis, gender differences were found in all parameters of knee surface. It seemed that males had larger resected knee surfaces than females in all dimensions. To describe the geometric shape of the resected surface, we introduced the following parameters. The aspect ratio was defined as the ratio of anteroposterior length and mediolateral width. Asymmetry ratio was defined as the ratio of medial and lateral anteroposterior length. A significantly higher femoral aspect ratio was found in the cases receiving Genesis-II prosthesis (p < 0.001), indicating a lower femoral mediolateral width is given anteroposterior length. No gender difference was found in tibial parameters in any prosthesis system. In linear regression of knee parameters on age, gender, height, weight, and other parameters in the same surface, the only influence factor confirmed by partial correlation in all three different prosthesis systems was the gender of femoral mediolateral width (Table 2). Height was the only factor in the linear regression of femoral anteroposterior length in the Genesis-II group and was confirmed by partial correlation in the Scorpio NRG group (p < 0.001), but it was excluded in the regression of the NexGen LPS group. Close relationships were identified between morphological parameters of resected tibiae. Such connection was not found in femur. The anthropometry results of knee prostheses were shown in Table 3.

**Table 1:** Anthropometry parameters of cases receiving different knee prosthesis

Parameter	Genesis-II			Scorpio NRG			NexGen LPS		
	Male (mm)	Female (mm)	P value	Male (mm)	Female (mm)	P value	Male (mm)	Female (mm)	p value
fosteo	10.45 ± 2.50	9.75 ± 2.69	0.078	7.51 ± 3.86	7.13 ± 3.78	0.602	10.71 ± 1.52	10.02 ± 2.06	0.109
fAP	45.66 ± 3.46	43.71 ± 5.92	< 0.001	45.51 ± 3.13	42.28 ± 3.91	< 0.001	48.24 ± 6.80	44.12 ± 7.38	< 0.001
fML	77.88 ± 3.94	69.89 ± 4.47	< 0.001	75.71 ± 4.01	68.48 ± 3.08	< 0.001	79.07 ± 2.88	69.68 ± 3.77	< 0.001
fAP/fML	0.59 ± 0.05	0.63 ± 0.11	< 0.001	0.60 ± 0.03	0.62 ± 0.06	0.018	0.61 ± 0.08	0.63 ± 0.10	0.308
tosteo	9.01 ± 2.45	8.53 ± 2.58	0.013	7.53 ± 3.56	7.00 ± 3.66	0.437	9.83 ± 1.61	9.35 ± 1.92	0.270
tMAP	52.65 ± 4.11	47.65 ± 4.06	< 0.001	50.89 ± 3.32	47.56 ± 4.12	< 0.001	52.81 ± 4.09	47.63 ± 2.59	< 0.001
tLAP	47.96 ± 3.62	43.33 ± 2.69	< 0.001	46.10 ± 2.64	42.54 ± 2.52	< 0.001	48.14 ± 2.85	43.05 ± 2.61	< 0.001
tML	77.64 ± 3.83	70.81 ± 4.30	< 0.001	76.04 ± 5.96	70.50 ± 4.12	< 0.001	78.12 ± 8.15	70.72 ± 3.24	< 0.001
tMAP/tLAP	1.10 ± 0.10	1.10 ± 0.08	0.970	1.10 ± 0.05	1.12 ± 0.10	0.218	1.10 ± 1.10	1.11 ± 0.06	0.747
tMAP/tML	0.68 ± 0.05	0.67 ± 0.06	0.459	0.67 ± 0.04	0.68 ± 0.07	0.549	0.69 ± 0.11	0.67 ± 0.03	0.667
tLAP/tML	0.62 ± 0.04	0.61 ± 0.04	0.321	0.61 ± 0.05	0.61 ± 0.05	0.698	0.62 ± 0.09	0.61 ± 0.03	0.488

**Table 2:** Multiple linear regression and partial correlation analysis of morphological parameters on demographical factors and parameters on the same surface

Variable	Genesis-II			Scorpio NRG			NexGen LPS		
	Standardized coefficients (beta)	P value	Pcorr value	Standardized coefficients (beta)	P value	Pcorr value	Standardized coefficients (beta)	p value	Pcorr value
fAP									
Age	N/A			N/A			N/A		
Gender	N/A			N/A			N/A		
Height	0.190	< 0.001		0.213	< 0.001	< 0.001	N/A		
Weight	N/A			N/A			N/A		
fML	N/A			0.250	0.001	0.001	0.544	< 0.001	N/A
fML									
Age	N/A			N/A			N/A		
Gender	6.298	< 0.001	< 0.001	5.406	< 0.001	< 0.001	6.851	< 0.001	< 0.001
Height	0.169	< 0.001	< 0.001	0.159	0.001	0.001	N/A		
Weight	N/A			N/A			0.141	< 0.001	< 0.001
fAP	N/A			0.208	0.002	0.002	0.142	0.001	0.001
fAP/fML									
Age	N/A			N/A			N/A		
Gender	− 0.042	< 0.001		N/A			N/A		
Height	N/A			N/A			N/A		
Weight	N/A			N/A			N/A		
tMAP									
Age	N/A			N/A			N/A		
Gender	0.962	0.043	0.043	N/A			1.739	0.039	0.039
Height	N/A			N/A			N/A		
Weight	N/A			N/A			0.060	0.020	0.020
tLAP	0.567	< 0.001	< 0.001	0.452	< 0.001	< 0.001	0.261	0.010	0.010
tML	0.207	< 0.001	< 0.001	0.252	< 0.001	< 0.001	0.174	0.006	0.006
tLAP									
Age	N/A			N/A			N/A		
Gender	0.937	0.005	0.005	1.772	< 0.001	< 0.001	2392	0.002	0.002
Height	0.114	< 0.001	< 0.001	N/A			N/A		
Weight	N/A			0.040	0.022	0.022	N/A		
tMAP	0.238	< 0.001	< 0.001	0.158	0.001	0.001	0.245	0.005	0.005
tML	0.198	< 0.001	< 0.001	0.171	< 0.001	< 0.001	0.193	0.001	0.001
tML									
Age	N/A			N/A			N/A		
Gender	3.034	< 0.001	< 0.001	N/A			N/A		
Height	N/A			0.255	< 0.001	< 0.001	0.225	0.003	0.003
Weight	0.035	0.022	0.022	N/A			N/A		
tMAP	0.228	< 0.001	< 0.001	0.266	0.002	0.002	0.412	0.003	0.003
tLAP	0.505	< 0.001	< 0.001	0.531	< 0.001	< 0.001	0.496	0.001	0.001
tMAP/tLAP									
Age	N/A			N/A			N/A		
Gender	N/A			N/A			N/A		
Height	N/A			N/A			N/A		
Weight	N/A			N/A			N/A		
tML	N/A			N/A			N/A		
tMAP/tML									
Age	N/A			N/A			N/A		
Gender	N/A			N/A			N/A		
Height	N/A			N/A			N/A		
Weight	N/A			N/A			N/A		
tLAP	0.002	0.001		N/A			N/A		
tLAP/tML									
Age	N/A			N/A			N/A		
Gender	N/A			N/A			N/A		
Height	0.001	0.008		N/A			N/A		
Weight	N/A			N/A			N/A		
tMAP	N/A			N/A			N/A		

Pcorr value is the P value of partial correlation analysis. N/A refers to exclusion from linear regression model

**Table 3:** Anthropometry parameters of prosthesis systems

Femur size	Genesis-II			Femur size	Scorpio NRG		
	fAP (mm)	fML (mm)			fAP (mm)	fML (mm)	
1#	30.33	55.84		3#	35.49	57.49	
2#	34.57	58.72		4#	37.50	59.60	
3#	38.13	62.72		5#	39.60	62.63	
4#	42.12	67.24		6#	41.47	65.04	
5#	44.82	70.73		7#	44.12	67.82	
6#	48.27	73.34		8#	46.55	69.91	
				9#	48.70	72.44	
				11#	53.65	77.38	

Tibia size	tMAP (mm)	tLAP (mm)	tML (mm)	Tibia size	tMAP (mm)	tLAP (mm)	tML (mm)
1#	42.00	37.50	60.02	3#	38.00	38.00	60.81
2#	44.50	40.50	64.95	4#	39.00	39.00	63.34
3#	47.50	42.50	68.00	5#	41.00	41.00	65.98
4#	49.30	45.00	71.01	6#	42.00	42.00	68.71
5#	51.00	47.00	74.02	7#	44.00	44.00	71.42
6#	53.50	48.30	77.00	9#	48.00	48.00	76.61
				11#	51.00	51.00	81.98

### Prothesis coverage simulation

The two systems with larger case sizes, Genesis-II and Scorpio NRG, were included in the coverage simulation (Fig. 2). These prostheses covered more than 93% of cases in at least one parameter. For femoral surface, Genesis-II and Scorpio NRG had little difference in the percentage of complete coverage (31.88% vs. 38.60%) and relative coverage (61.58% vs. 61.40%). Scorpio NRG had no record in no coverage, while the percentage of Genesis-II was 6.53%. The performance of medial tibia coverage was almost the same. Genesis-II had better lateral tibia complete coverage (61.19% vs. 27.49%). Scorpio took advantage in relative coverage (69.01% vs. 32.48%) and no coverage (3.51% vs. 6.34%) (Table 3). According to previous results, only femoral morphology had significant gender differences. In scatter plots of femoral parameters, male cases tend to take the upper right corner, reflecting the bigger size than females (Fig. 2a, d). Lower complete coverage, higher relative coverage, and no coverage percentages were seen in stimulation, suggesting better coverage for females than males (Table 4).

**Table 4:** The simulated prosthesis coverage of knee resected surface

Parameters	Genesis-II			Scorpio NRG		
	Complete coverage	Relative coverage	No coverage	Complete coverage	Relative coverage	No coverage
fAP-fML	31.88% (161/505)	61.58% (311/505)	6.53% (33/505)	38.60% (66/171)	61.40% (105/171)	0
Male	10.58% (11/104)	68.27% (71/104)	21.15% (22/104)	11.11% (4/36)	88.89% (32/36)	0
Female	37.41% (150/401)	59.85% (240/401)	2.74% (11/401)	45.93% (62/135)	54.07% (73/135)	0
tMAP-tML	57.62% (291/505)	35.64% (180/505)	6.73% (34/505)	60.82% (104/171)	39.18% (67/171)	0
tLAP-tML	61.19% (309/505)	32.48% (164/505)	6.34% (32/505)	27.49% (47/171)	69.01% (118/171)	3.51% (6/171)

## Discussion

The initial design of knee prostheses had only one implant size following the average of some samples [25]. Later, detailed X-ray and cadaveric anthropometries suggested multiple sizes of prostheses to match natural knee geometries [5, 7]. With the development of operation techniques and surgical instruments, improving the fitness between the prosthesis and the resected bone surface has been a new route achieving “forgotten knee”. Gender has been regarded as an important factor affecting knee morphology.

There was number of studies demonstrating knee morphological diversity between gender. In an intraoperative anthropometric study of Chinese tibiae, significant larger medial and lateral AP were found in males [21]. In tomography measurement of Chinese femurs, narrower ML and shorter medial AP were found when normalized by lateral AP [26]. A similar study of Iranian tibiae found narrower ML in females [27]. Radiographical studies of Indian, Thai, Malay, and Turkish populations agreed with a larger knee in males [19, 28–30]. However, some researchers adjusted other demographical factors like height and weight in the evaluation of gender differences in knee quadriceps angle (Q angle). After multiple linear regression, they found that height rather than gender had a more significant influence [31]. In the present study, we used a similar method adjusting confounding factors, which was taken into consideration in few studies. Our anthropometric results fully supported gender difference only in ML width of the femur. Such differences were influenced by correlations between morphological parameters for tibiae and height diversity for the femur. The significant difference of aspect ratio was only found in one of three kinds of prosthesis for femur, provided limited support to narrower femur surface for females in given AP length. Interestingly, according to coverage calculation of different gender, we found lower coverage in males than females, which may explain the unsatisfied results of gender-specific prostheses. These designs tried to introduce smaller sizes with narrower ML widths to improve coverage. Meta-analysis of 1120 TKA cases confirmed a lower overhang rate, but the evidence was insufficient for the improvement of outcome. Some researchers reported optimistic reports [32], and more studies found no benefit in follow-up [17, 18]. The regression results described a larger femoral surface for males. Maybe the initial direction of design modification should be reversed. Larger femoral prostheses with wider ML width could be another choice to improve coverage. More clinical observations and trials are required for further investigation.

**Fig. 3:**
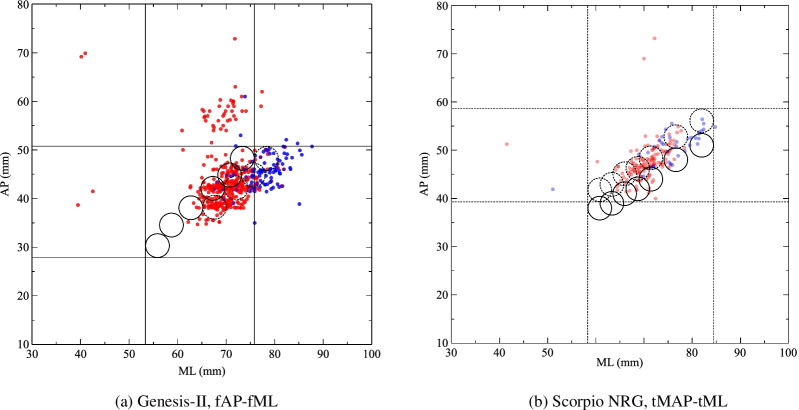
Two possible ways of prosthesis modification. **a** Introduction of new sizes having wider mediolateral width (shown as dashed circles) increases the femoral coverage. **b** Increase medial tibial anteroposterior length of Scorpio NRG using average asymmetry ratio (tMAP/tLAP) of Genesis-II, the new plot area has higher tibial coverage (shown in dashed circles and lines)

Early knee anthropometries were mainly finished in western countries, so the sizes of prothesis were designed for Caucasian populations. The diversity of knee morphology in different ethics has been proved by many researchers. A recent study compared Asians and Dutch Caucasians in Indonesia. Measurement of radiography found that Caucasians had large AP and ML size in both femur and tibia, The ML/AP ratios of both sides were higher in Asians, suggesting a wider surface in given AP [33]. Kim et al. conducted a systematic review of knee morphology based on 30 studies (9050 knees); the result showed that white people had larger knee sizes with reduced ML/AP ratio [9]. The current study supported their findings. In AP-ML plots, the case points tended to distribute on the right side of circles representing prosthesis sizes, indicating a larger ML in given AP (Fig. 2a, d). Extra improvement of the knee prosthesis is necessary to fit the different knee morphology of Chinese and other Asian populations. The introduction of more sizes of prostheses is a potential direction of modification. In the current study, the femoral prosthesis series of Genesis-II and Scorpio NRG covered almost the same range in AP (17.94 mm vs. 18.46 mm) and ML (17.50 mm vs. 19,89 mm). Genesis-II has six femoral sizes and Scorpio NRG has eight, and the percentage of complete coverage was a higher group with more sizes (38.60% vs. 31.88%). Under a given range of the parameter, the more sizes a TKA system gets, the wider range it may cover and the fitness of diverse knees could be better (Fig. 3a). Change of prosthesis shape was another possible way. For the tibial plateau, there were two design principles: symmetry plateau has the same tMAP and tLAP, and asymmetry plateau made tMAP a bit larger. The intraoperative anthropometry of Miyatake et al. reported better coverage of asymmetry plateau via a semi-quantitative method [11]. The computer analysis of tomography data from Clary et al. found similar coverage among two symmetry and two asymmetry plateaus [34]. The current study showed that asymmetry design (Genesis-II) had higher (for lateral) or similar (for medial) complete coverage than symmetry design (Scorpio NRG) with fewer prosthesis sizes. Asymmetry plateau with more prosthesis sizes could be an ideal solution (Fig. 3b). The design and production of new prosthesis sizes and shapes is time and economic-consuming, so more detailed analysis and simulations are required for these design changes.

There are some limitations to this study. First, it is a single-center observational study, more prospective studies with larger sample sizes are yet to be carried out. Then, the intraoperative manual measurement has an unavoidable error, which could affect the accuracy of coverage. Third, post-operation radiology measurement of prosthesis coverage is also needed for better analysis of actual prosthesis position. With more data from future studies, it is possible to provide more accurate data for the development of knee prostheses for Chinese and other Asian populations.

## Conclusions

In the current study, we reported the morphological characters of the distal femur and proximal tibial resected surface in the Chinese population. After adjusting confounding factors, only gender difference on femoral mediolateral width was fully supported in knee morphology. Males tended to have lower femoral prosthesis coverage, so enlargement of sizes could be another direction for gender-specific prosthesis design. The Western-designed prostheses showed well coverage of most Chinese knee surfaces, but the percentage of complete coverage was too low for a better outcome. Introduction of more prosthesis sizes and improvement of prosthesis shape are possible ways to improve the coverage and clinical outcome of TKA in Chinese population.

## Data Availability

The datasets used and/or analyzed during the current study are available from the corresponding author on reasonable request.

## Abbreviations

AP: Anteroposterior length
BMI: Body mass index
fAP: Femoral anteroposterior length
fML: Femoral mediolateral width
fosteo: Femoral osteotomy thick
ML: Mediolateral width
TKA: Total knee arthroplasty
tLAP: Tibial lateral anteroposterior length
tMAP: Tibial medial anteroposterior length
tML: Tibial mediolateral width
tosteo: Tibial osteotomy thick
